# Recovery of the histamine H_3_ receptor activity lost in yeast cells through error-prone PCR and in vivo selection

**DOI:** 10.1038/s41598-023-43389-z

**Published:** 2023-09-26

**Authors:** Ayami Watanabe, Ami Nakajima, Mitsunori Shiroishi

**Affiliations:** Department of Biological Science and Technology, Tokyo University of Science, 6-3-1 Niijuku, Katsushika-ku, Tokyo, 125-8585 Japan

**Keywords:** G protein-coupled receptors, Protein design, Receptor pharmacology, Applied microbiology, Protein engineering

## Abstract

G protein-coupled receptors (GPCRs) are the largest protein family in humans and are important drug targets. Yeast, especially *Saccharomyces cerevisiae*, is a useful host for modifying the function and stability of GPCRs through protein engineering, which is advantageous for mammalian cells. When GPCRs are expressed in yeast, their function is often impaired. In this study, we performed random mutagenesis using error-prone PCR and then an in vivo screening to obtain mutants that recovered the activity of the human histamine H_3_ receptor (H_3_R), which loses its signaling function when expressed in yeast. Four mutations with recovered activity were identified after screening. Three of the mutations were identified near the DRY and NPxxY motifs of H_3_R, which are important for activation and are commonly found in class A GPCRs. The mutants responded exclusively to the yeast YB1 strain harboring G_i_-chimera proteins, showing retention of G protein specificity. Analysis of one of the mutants with recovered activity, C415R, revealed that it maintained its ligand-binding characteristics. The strategy used in this study may enable the recovery of the activity of other GPCRs that do not function in *S. cerevisiae* and may be useful in creating GPCRs mutants stabilized in their active conformations.

## Introduction

G protein-coupled receptors (GPCRs) are the largest and most diverse group of proteins among more than 800 members of the human genome^1^. This receptor group receives various ligands, such as light, chemical substances, peptides, and proteins, and transmits signals to cells via a heterotrimeric G protein. GPCRs are involved in many diseases because they mediate various physiological functions, such as vision, smell, taste, neurotransmission, and endocrine and immune responses. As a result, one-third of currently approved drugs target GPCRs^2^. Understanding the molecular function of GPCR is thus important for drug discovery^3^. In addition to X-ray crystallography, single-particle analysis using cryo-electron microscopy has recently been developed for such characterization^4^. Nuclear magnetic resonance techniques and other spectroscopic analyses have also been developed to elucidate the dynamics of GPCR function^5^. However, a highly purified target receptor protein at the milligram scale is required for structural and physicochemical analyses. Most structurally elucidated GPCRs have been stabilized for large-scale preparation; however, stabilization often fixes the conformation of the receptor in its active or inactive form^6–10^. Therefore, stabilization of GPCRs while retaining their functionality remains a challenge.

The histamine receptor is a member of the GPCR family that receives histamine, a bioactive amine. Histamine receptors include H_1_ and H_2_, which have low affinities for histamine, and H_3_ and H_4_, which have high affinities for histamine. The histamine H_3_ receptor (H_3_R) is mainly expressed in the central nervous system and suppresses the release of bioactive substances at the synapses^11,12^. H_3_R is involved in cognitive function, and its antagonism is associated with the therapeutic outcomes of conditions, such as attention deficit hyperactivity disorder (ADHD), schizophrenia, Alzheimer's disease, and narcolepsy^12^. According to recent studies, H_3_R agonists are effective against insomnia, obsessive–compulsive disorders, and cardiorenal damages^13–15^. The structure of the H_1_ receptor in both the active and inactive states has been clarified^16,17^. In addition, the crystal structure of H_3_R in its inactive form was recently revealed; however, the structure of the active state has not been elucidated^18^.

Yeasts, especially *Saccharomyces cerevisiae*, are commonly used in GPCR studies as platforms for selecting stable variants and functional analyses of GPCRs. *S. cerevisiae* grows rapidly and is easy to handle, thereby enabling the production and evaluation of numerous GPCR variants^19^. Although mammalian cells have many endogenous receptors and signaling pathways, yeast has only one signal transduction system via the MAPK pathway (pheromone signaling pathway) by Ste2, a yeast GPCR. This pathway finally activates Ste2 responsive genes, such as *FUS1* and *FUS2*, which are required for mating. The signal transduction system in yeast is simpler than that in mammalian cells, making it easier to analyze coupling events between GPCRs and trimeric G proteins^20^. To date, *S. cerevisiae* strains with a chimeric protein, in which the five C-terminal residues of yeast Gpa1 have been replaced with the human Gα, have been produced; these strains have been used in functional analysis of various GPCRs and deorphanization^21–23^. By introducing genes involved in amino acid synthesis, such as *HIS3*, downstream of the *FUS1* promoter, to the yeast strain with the original *HIS3* gene deleted, auxotrophy-based screening can be performed according to the signal strength. By combining yeast strains with molecular evolution techniques, an artificial GPCR that reacts with the designer ligand in vivo was produced via auxotrophy-based screening^24^. Attempts were also made to identify the residues important for receptor function using a selection system with a *FUS2-CAN1* reporter and canavanine^25^.

Currently, signal transduction has been confirmed in *S. cerevisiae* for approximately 50 mammalian GPCRs. However, expressing GPCRs in yeast and constructing a signal transduction assay system are generally difficult tasks^21,26^. In our study, human H_3_R expressed in a *S. cerevisiae* strain harboring a human Gα_i_ chimera did not exhibit signal transduction activity. Random mutations were introduced into the receptor gene using error-prone PCR, and the receptor library was selected in vivo using an agonist (histamine)-containing selective medium, resulting in mutants with recovered histamine signal transduction activity. The discovered mutations were located at positions that have not been reported to be involved in GPCRs.

## Materials and methods

### Yeast strains and growth media

The yeast strains, YB1 (also known as MPY578t5), YB11 (MPY578q5) and YB14 (MPY578s5)^27^, were kindly provided by Professor Bryan Roth’s laboratory at the University of North Carolina, Chapel Hill (North Carolina, USA). The -Ura and -Ura-His dropout supplements were prepared according to a published formulation^28^. YPAD medium (20 g/L peptone, 10 g/L yeast extract, 2% glucose, 20 mg/L adenine sulfate) was used for non-selective applications, while synthetic complete (SC) medium without uracil (-Ura/SC; 1.7 g/L yeast nitrogen base without amino acids, 5 g/L ammonium sulfate, 1.92 g/L -Ura dropout supplement, and 2% glucose) or SC medium without uracil and histidine (-Ura-His/SC; 1.7 g/L yeast nitrogen base without amino acids, 5 g/L ammonium sulfate, 1.92 g/L -Ura-His dropout supplement, and 2% glucose) was used for selective applications. The *Escherichia coli* strain, DH5α, was grown in LB medium containing 100 μg/mL ampicillin for plasmid preparation.

### Ligands

Histamine hydrochloride (# 085-03554) was purchased from FUJIFILM Wako chemicals (Osaka, Japan). Imetit (# 0729), iodophenpropit (#0779), JNJ-5207852 (#4240), BF2649 (#3743), clobenpropit (#0752), and A331440 (#4697) were purchased from Tocris Biosciences (Bristol, UK).

### Plasmids

Plasmid p416GPD was kindly provided by Professor Bryan Roth. A codon-optimized cDNA of the full-length histamine H_3_ receptor (H_3_R_FL), which has an N11Q mutation that prevents glycosylation, was kindly provided by Professor Iwata’s laboratory at Kyoto University (Kyoto, Japan). A series of genes, including the TEV protease cleavage site, enhanced green fluorescent protein (GFP), and histidine tag, were integrated downstream of the GPD promoter in the p416GPD plasmid, resulting in plasmid p416GPD_GFP. The H_3_R_FL or H_3_R variants with the third intracellular loop removed from 237 to 340 (H_3_R_i3d) were integrated immediately before the TEV site of the p416GPD_GFP plasmid, resulting in an expression plasmid in which GFP was fused to the C-terminus of the receptor (p416GPD_H_3_R_FL-GFP or p416GPD_H_3_R_i3d-GFP). The *H*_*3*_*R_i3d-GFP* gene was integrated into the pPIC9K plasmid (pPIC9K_H_3_R_i3d-GFP), a *Pichia pastoris* plasmid, to prevent template-derived colonies when transforming DNA fragments of error-prone PCR into *S. cerevisiae*.

### Random mutagenesis and generation of the yeast library

Random mutations were introduced via error-prone PCR using a GeneMorph II Random Mutagenesis Kit (Agilent Technologies). The reactions were performed according to the manufacturer’s instructions. Approximately 4.8 μg of template pPIC9K_H_3_R_i3d-GFP (~ 11.3 kb) was used per reaction volume (50 μL). This amount corresponds to approximately 420 ng of target DNA (H_3_R_i3d; ~ 1 kb) per reaction. Five reaction solutions (250 μL in total) were prepared for each transformation. To amplify the *H*_*3*_*R_i3d* gene for random mutation, a forward primer 81_GPD_F (5′-AGTTTCGACGGATTCTAGAACTAGGGATCCATGGAAAGAGCTCCA-CCAGATGG-3′) and a reverse primer 81_YHR_R (5′-AAATTGACCTTGAAAA-TATAAATTTTCCCCCTTCCAACAATGTTCCAAAGAAGAATG-3′) were used. The primers contained an overlapping region in the p416GPD_GFP vector for gap repair recombination cloning. The reaction was carried out in 30 cycles at an annealing temperature of 50 °C. The desired fragments were confirmed using electrophoresis.

After random mutagenesis, both the SmaI-digested p416GDP_GFP and DNA fragment of H_3_R_i3d were co-transformed into the yeast YB1 strain. Briefly, an overnight culture of YB1 in YPAD medium was subcultured in 500 mL of fresh YPAD medium to an OD_600_ of 0.6 at 30 °C. The cells were recovered from the culture medium via centrifugation at 3000 rpm for 5 min using a swinging-bucket rotor, washed with sterile water, and then suspended in 4 mL of 0.1 M LiAc/TE. Single-strand carrier DNA (10 mg), 20 μg SmaI-digested p416GPD_GFP, 250 μL of random mutagenesis reaction mix, and 30 mL of 40% PEG3350/0.1 M LiAc/TE were added to the cell suspension, which was then stirred well using a vortex mixer and then incubated for 30 min with shaking at 30 °C. DMSO (3.5 mL) was added to the cell suspension, and heat shock was performed at 42 °C for 25 min. The cells were then harvested and washed once with water. The cell pellet was resuspended in 3 mL of water and plated on 15 × 15 cm plates of -Ura-His/SC medium containing 10 mM 3-AT and 100 μM histamine. The plates were incubated at 30 °C for 4 days. A portion of the cell suspension was plated on a -Ura/SC medium and incubated at 30 °C for 3 days to determine the colony-forming units per amount of plasmid (cfu/μg).

### Recovering plasmid DNA from yeast

Yeast colonies were inoculated in 5 mL of -Ura/SC medium, and left to grow for 18–20 h at 30 °C with shaking. The cells were collected via centrifugation and the supernatant was discarded. The cell pellet was resuspended in 500 μL of 1 M sorbitol solution, and 20 μL of 12.5 mg/mL Zymolyase 20 T (Nacalai Tesque, Japan) was added. The cell suspension was incubated at 37 °C for 40 min and then centrifuged at 12,000*g* for 1 min at 22–25 °C. After removal of the supernatant, the cell pellet was resuspended in 200 μL of mP1 solution from the FastGene Plasmid Mini Kit (NIPPON Genetics, Japan). A 150-μL volume of acid-washed glass beads (Sigma) was added, and the cells were disrupted by vortexing at 4 °C for 2 min. The disrupted cell suspension was transferred to a clean tube and 200 μL of mP2 solution was added. Subsequent operations were performed according to the manufacturer’s instructions for plasmid extraction from *E. coli*.

### Ligand-concentration dependent growth assay

Yeast colonies were inoculated in 5 mL of -Ura/SC medium in test tubes, and grown at 30 °C for 20–22 h at 160 rpm in a shaking incubator. The cells were collected via centrifugation at 8000*g* for 3 min, and the supernatant was discarded. The cells were then resuspended in sterile water and collected via centrifugation at 8000*g* for 3 min. After the supernatant was discarded, the cells were then resuspended in -Ura-His/SC medium at an OD_600_ of 1. Thereafter, the cells were diluted to an OD_600_ of 0.02 in 500 μL of the -Ura-His/SC medium with 10–40 mM 3-AT and a fivefold dilution series of ligands in a 96-well deep well plate, and cultured at 25 °C for 70 h at 1450 rpm in an M/BR-022UP shaker (TAITEC, Japan). The culture (100 μL) was transferred to a 96-well microplate, and the OD_595_ was measured using an iMark micro plate reader (Bio-Rad, USA). Curve fitting was performed using GraphPad Prism 6.0 (GraphPad Software, San Diego, CA, USA).

### Measuring whole-cell GFP fluorescence

Yeast cells cultured in 500 μL -Ura/SC medium in 96-well deep well plates were collected at 3000*g* for 30 min at 4 °C using a microplate swinging-bucket rotor. The supernatant was then removed via suction using an aspirator. The cells were resuspended in 160 μL/well of the resuspension buffer (50 mM Tris–HCl pH 7.5, 5 mM EDTA, 10% glycerol, 0.12 M sorbitol) with the complete protease inhibitor EDTA-free cocktail (Merck). The fluorescence of a 20-μL cell suspension was measured in a small volume 384-well microplate (Greiner Bio-One, Frickenhausen, Germany) at an excitation wavelength of 490 nm and a detection wavelength of 525 nm using a 515 nm cutoff filter and a Spectra Max Gemini XPS fluorescence plate reader (Molecular Devices, USA).

### Yeast membrane preparation

Colonies were inoculated in 10 mL of -Ura/SC medium in test tubes and grown at 30 °C for 20–22 h at 160 rpm in a shaking incubator. The cells were diluted to an OD_600_ of 0.12 in a fresh 1-L medium of -Ura/SC in a Tunair flask, and grown at 30 °C for 20 h at 160 rpm. The cells were collected via centrifugation at 6000*g* for 10 min at 4 °C, and then resuspended in 12 mL of the resuspension buffer (50 mM Tris–HCl pH 7.5, 5 mM EDTA, 10% glycerol, 0.12 M sorbitol) with 1 × concentration of the complete protease inhibitor EDTA-free cocktail (Merck). For cell disruption, the cells were mixed with 15 mL of 0.5 mm glass beads and then disrupted using a vortex mixer that operated for 12 cycles (1 min of disruption and 8 min of incubation on ice). Undisrupted cells and cell debris were pelleted via centrifugation at 3000*g* for 30 min at 4 °C, and the supernatant, including the yeast membrane, was collected in microtubes. The membrane was pelleted via centrifugation at 100,000*g* for 30 min at 4 °C using a Himac CS100FNX Micro Ultracentrifuge (Eppendorf-Himac, Japan). The membrane pellet was resuspended in membrane buffer (50 mM Tris–HCl pH 7.5, 120 mM NaCl, 20% glycerol) with 1 × concentration of the complete protease inhibitor EDTA-free cocktail. The protein concentration of the membrane suspension was determined using the TaKaRa BCA Protein Assay Kit (Takara Bio, Japan).

### In-gel fluorescence

The membrane suspensions of the wild type and each mutant were adjusted to a protein concentration of 5 mg/mL. Fifteen microliters of the membrane suspension (5 mg/mL) was mixed with an equal volume of 2 × sample buffer (50 mM Tris–HCl pH 7.5, 5 mM EDTA, 5% β-mercaptoethanol, 5% glycerol, 4% SDS, 0.02% bromophenol blue, 2 × concentration of complete EDTA-free protease inhibitor cocktail), and 20 μL of the mixture was applied to the SDS-PAGE gel of the Tris–glycine buffer system without boiling the sample. One microliter of the Benchmark Fluorescent Protein standard (Thermo Fisher Scientific, USA) was applied to the gel as a molecular size marker. Electrophoresis was performed at 100 V and 4 °C. Fluorescence images were obtained using Typhoon FLA 7000 (GE Healthcare, USA).

### Radioisotope (RI)-labeled ligand binding assay

The membrane suspension, ^3^H-histamine (PerkinElmer, USA), and cold ligand (histamine) were diluted with the assay buffer (20 mM HEPES pH7.5, 150 mM NaCl). Briefly, 100 μL of the reaction mixture was dispensed in triplicate in a 96-well microplate. Membrane proteins (300 μg) were added to each well. The final concentrations of ^3^H-histamine were 200, 100, 50, 25, 12.5, 6.25, and 3.13 nM. To achieve non-specific binding, the experiment was performed in the presence of a cold ligand at a 1000-fold concentration of the hot ligand. The reaction mixture was incubated at room temperature (22–24 °C) for 1 h. The membrane was harvested on a glass fiber Filtermat B (PerkinElmer, USA) filter paper presoaked in 0.3% polyethylene imine using a FilterMate cell harvester (PerkinElmer, USA). The filter paper was washed with distilled water and dried. The solid scintillator, MeltiLex B/HS (PerkinElmer, USA), was melted on the filter paper. Radioactivity was detected using a Microbeta 2 system (PerkinElmer, USA). Specific binding was determined by subtracting nonspecific binding from total binding.

## Results

### H_3_R expressed in *S. cerevisiae* does not possess signaling activity

In this study, we used the engineered yeast strain, YB1, which possesses a chimeric yeast Gα protein, in which five C-terminal residues of Gpa1 are replaced with human Gα_i_. If a heterologously expressed GPCR couples with a chimeric protein, the yeast pheromone signaling pathway is activated. Of note, YB1 lacked the *HIS3* gene; therefore, it could not grow in histidine-depleted medium. When the expressed GPCR is activated by an agonist, the yeast pheromone signaling pathway is activated, which finally activates the *FUS1-HIS3* gene, resulting in cell growth in a histidine-depleted medium. The degree of cell proliferation depends on the strength of the signal (i.e., the agonist concentration).

**Figure 1:**
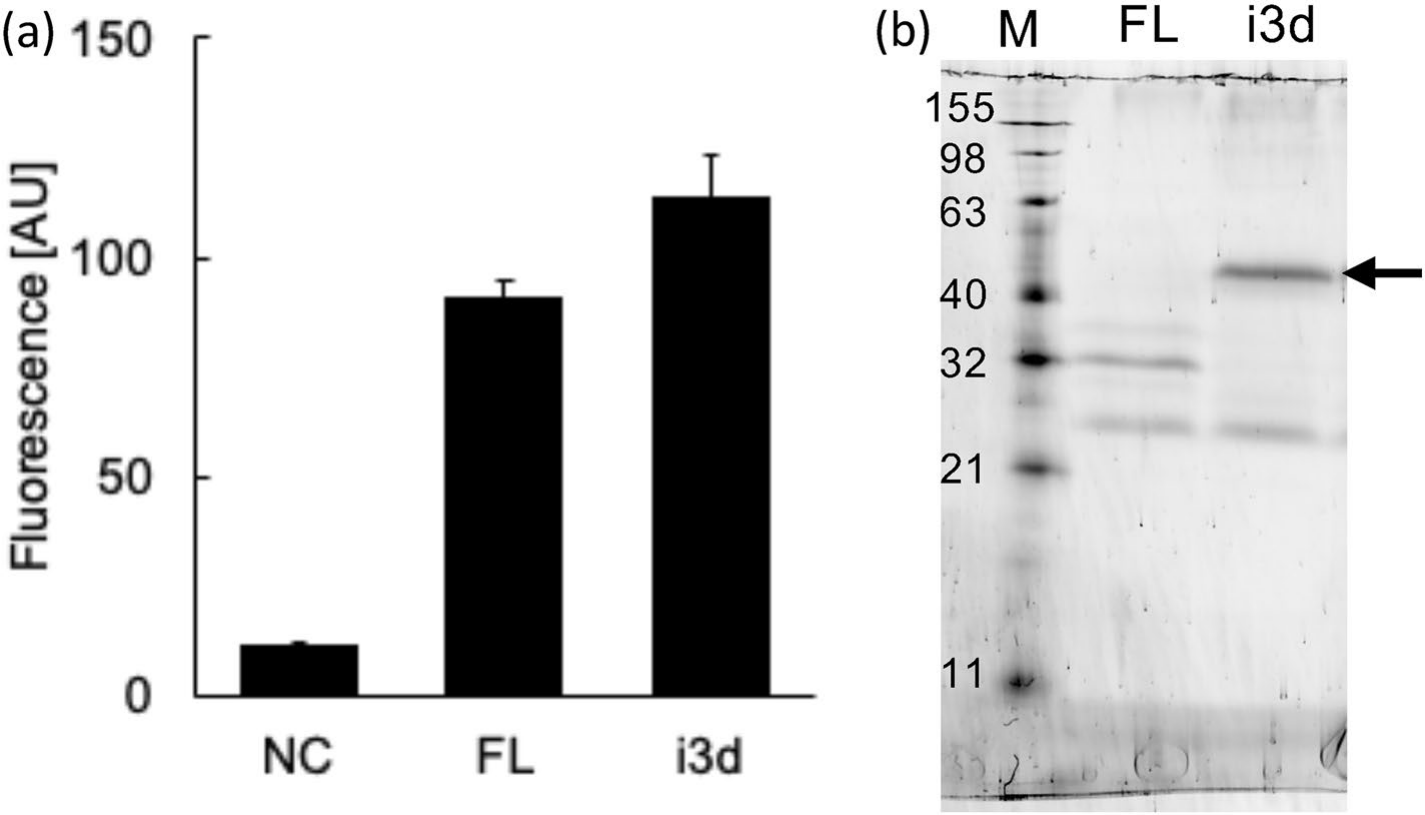
Expression of full-length H_3_R (FL) and the I3L-deleted H_3_R (i3d) in the YB1 strain of *S. cerevisiae*. NC is the YB1 strain transformed with empty p416GPD plasmid as a negative control. (**a**) Whole-cell GFP fluorescence intensity. Average ± SEM of eight colonies. (**b**) In-gel fluorescence after SDS-PAGE. Arrow indicates the band derived from H_3_R_i3d-GFP observed between 63 and 40 kDa. *M* molecular size marker.

The full-length H_3_R (H_3_R_FL) used in this experiment had an N11Q mutation introduced to avoid glycosylation via post-translational modifications. Glycosylation is important for receptor activity in certain GPCRs. However, Peng et al. confirmed the signal transduction activity of the H_3_R variant by deleting residues 1–26 at the N-terminus^18^. Therefore, the N11Q mutation did not significantly affect activity. The YB1 strain was transformed with the expression vector, H_3_R_FL containing GFP fused to the C-terminus of the receptor. After the transformants were cultured in -Ura/SC medium, an increase in GFP fluorescence was observed (Fig. 1a). In-gel fluorescence^29^ of the yeast membrane showed that bands were observed at a position approximately 32 kDa although the expected molecular mass of H_3_R_FL-GFP was approximately 78 kDa, which was considered to be due to receptor degradation (Fig. 1b). The band observed between 32 and 21 kDa, which was observed in all mutants, may be derived from a GFP released from the C-terminal of H_3_R by digestion. A ligand concentration-dependent growth assay using the natural agonist, histamine, was performed with the H_3_R_FL transformant; however, no growth was observed (data not shown).

As reported in a study that investigated muscarine M_1_, M_3_, and M_5_ receptors, deletion of a long intracellular third loop (I3L) can improve expression and coupling efficiency in *S. cerevisiae*^30^. Therefore, we constructed an H_3_R_i3d variant, in which the long I3L (from residues 237 to 340) was deleted. The GFP fluorescence of the transformant of H_3_R_i3d was similar to that of H_3_R_FL (Fig. 1a). In the in-gel fluorescence, a band that appeared to be derived from H_3_R_i3d-GFP (molecular mass of 67 kDa) was observed between 63 and 40 kDa (Fig. 1b, indicated by an arrow). In many cases, when electrophoresis is performed without boiling, the band representing membrane proteins is approximately 20% smaller than that representing their actual molecular mass^31^. However, ligand concentration-dependent growth was not observed (WT; Fig. 3a).

### Random mutagenesis and selection on the histamine plate

**Figure 2:**
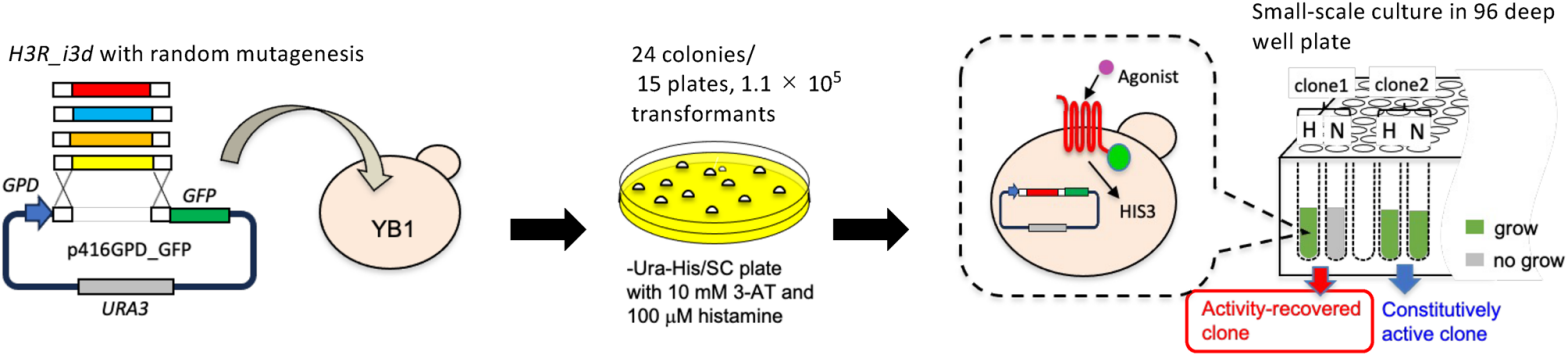
Schematic diagram of the screening experiment to obtain mutants with recovered activity.

**Figure 3:**
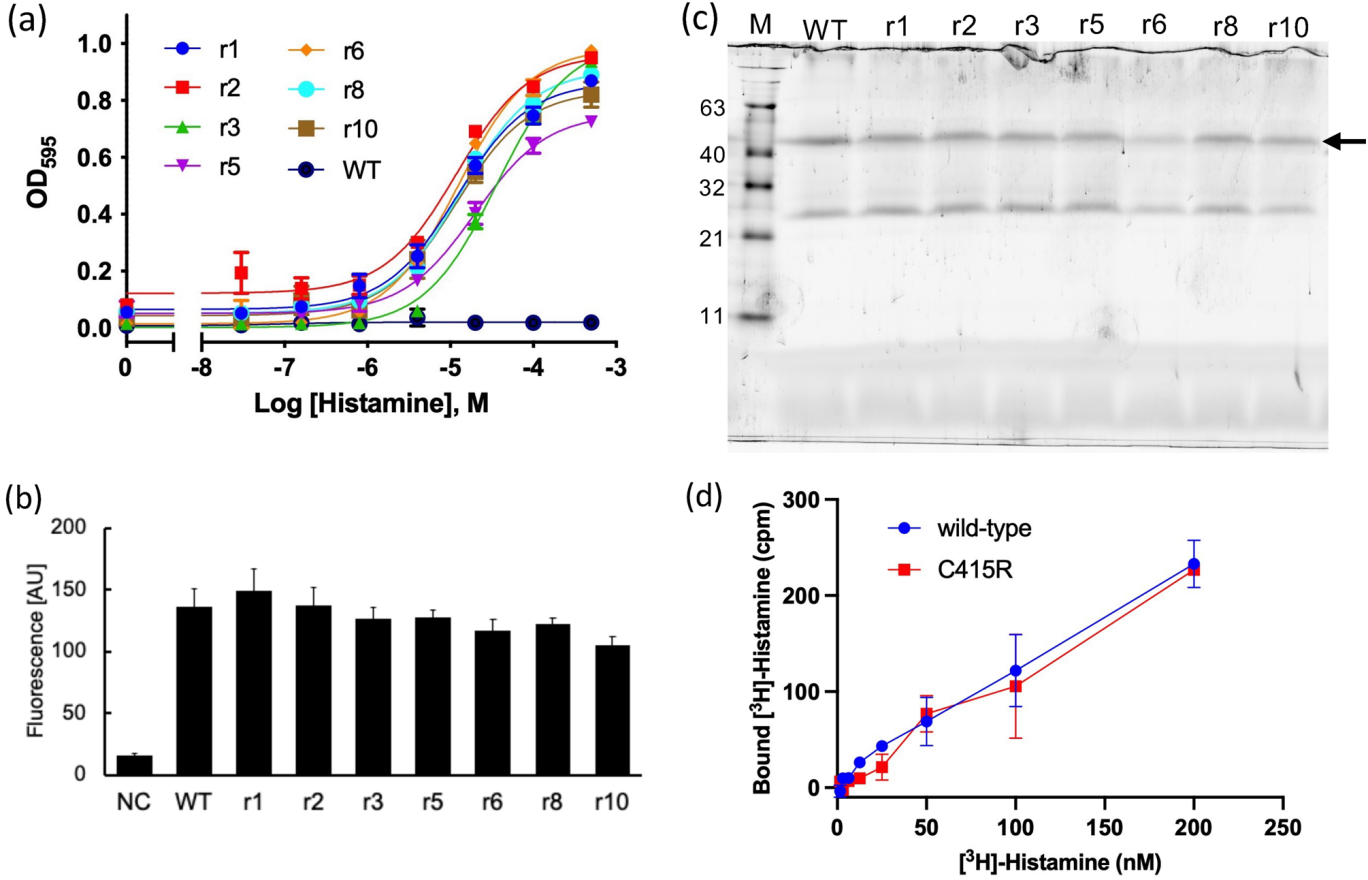
Characterization of the H_3_R mutants with recovered activity. (**a**) Agonist (histamine) concentration-dependent growth assay of the H_3_R clones (r1–r10) with recovered activity. Data shown here are representative of three independent experiments. Each experiment was performed in triplicate. The growth assay data were fitted to a Log (agonist) vs. response model. The mean EC_50_ ± SEM is shown in Table 1. (**b**) Whole-cell GFP fluorescence of the clones. Average ± SEM of eight colonies. (**c**) In-gel fluorescence after SDS-PAGE. Arrow indicates the band derived from H_3_R_i3d-GFP observed between 63 and 40 kDa. M; molecular size marker. (**d**) Ligand (^3^H-histamine) binding assay using the membrane of the wild-type and C415R. Specific binding was plotted. Each experiment was performed in triplicate.

The selection strategy for the H_3_R mutants is shown in Fig. 2. The YB1 strain was transformed with the SmaI-digested p416GPD_GFP plasmid and H_3_R-i3d via random mutagenesis using error-prone PCR. Based on the number of colonies on the -Ura/SC plate medium, the transformation efficiency was approximately 5200 cfu/μg per plasmid. After selection on -Ura-His/SC plate medium containing 10 mM 3-AT and 100 μM histamine, 24 colonies grew from approximately 1.1 × 10^5^ transformants. After these colonies were cultured in -Ura/SC medium overnight, the culture was transferred to -Ura-His/SC medium containing 10 mM 3-AT and 100 μM histamine or the medium without histamine in 96-well deep well plates, and then cultured at 25 °C for 48 h. Seven independent clones (r1, r2, r3, r5, r6, r8, and r10) were finally identified, which grew in medium with histamine but not in medium without histamine. All the clones showed histamine concentration-dependent growth (Fig. 3a). The EC_50_ values for histamine were 10–39 μM (Table 1). These clones contained one to four base substitutions in H_3_R_i3d (Table 2).

**Table 1:** Activation profile of the clones with recovered activity by the agonist histamine. EC_50_ values were obtained from the growth assays. The values are expressed as mean ± SD of three independent experiments.

Clone	EC_50_ (μM)
r1	10 ± 3
r2	12 ± 3
r3	38 ± 9
r5	39 ± 14
r6	14 ± 3
r8	14 ± 3
r10	25 ± 16

**Table 2:** Mutations found in the clones with recovered activity. The position (Ballesteros–Weinstein numbering) of the residue is shown as a superscript.

Clone	Mutations
r1	A39T^1.37^, H187L^ECL2^, S359Y^6.36^, V407A^7.48^
r2	F102L^ECL1^, C415R^7.56^
r3	L73M^2.43^
r5	A61T^1.59^, L117S^3.35^
r6	F193S^ECL2^
r8	C415R^7.56^
r10	V83I^2.53^, M156I^4.46^, F423L^8.54^

The integrity and activity of the receptor protein expressed in YB1 were analyzed in activity-recovered clones. The YB1 strain transformed with H_3_R_i3d (wild-type) or the activity-recovered clones was cultured in -Ura/SC (containing histidine) medium at 25 °C for 18 h, and the fluorescence was measured. No significant difference was found between wild-type and each clone in terms of GFP fluorescence intensity (Fig. 3b). In-gel fluorescence analysis of the cell membrane after SDS-PAGE revealed a band between 40 and 63 kDa for all mutants, similar to the wild-type H_3_R_i3d (Fig. 3c). A ligand-binding assay using ^3^H-histamine was performed using a membrane fraction prepared from the wild-type and clone r8 (C415R mutant). The dissociation constants (K_d_s) could not be determined due to lower binding to the amount of membrane used; however, similar specific binding was confirmed for the wild-type and clone r8 (Fig. 3d).

### Determination of mutations for the recovery of signaling activity

**Figure 4:**
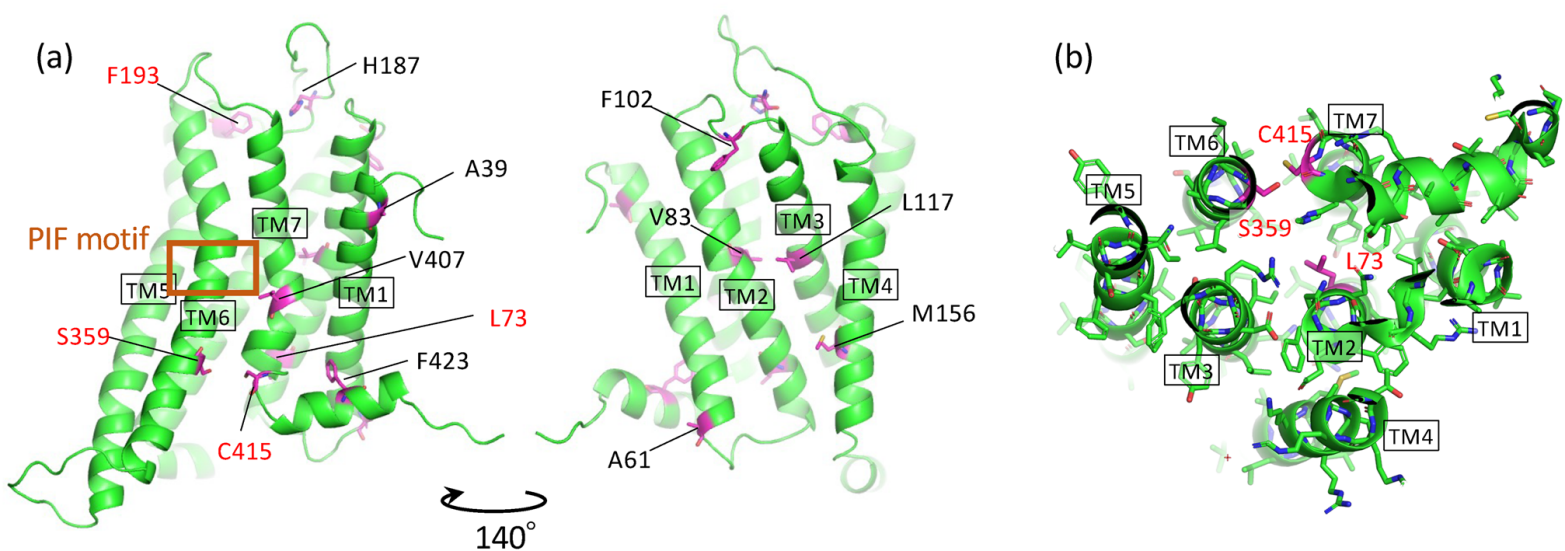
(**a**) Homology model of the H_3_R active-state built based on the H_1_R-G_q_ complex structure (PDBID: 7DFL) with the GPCRdb homology modeling pipeline, version 2019-03-14. The model was obtained from GPCRdb. (**b**) Crystal structure of H_3_R (PDBID: 7F61) viewed from the intracellular side. The mutations found in the activity-recovered clones are shown in magenta.

**Figure 5:**
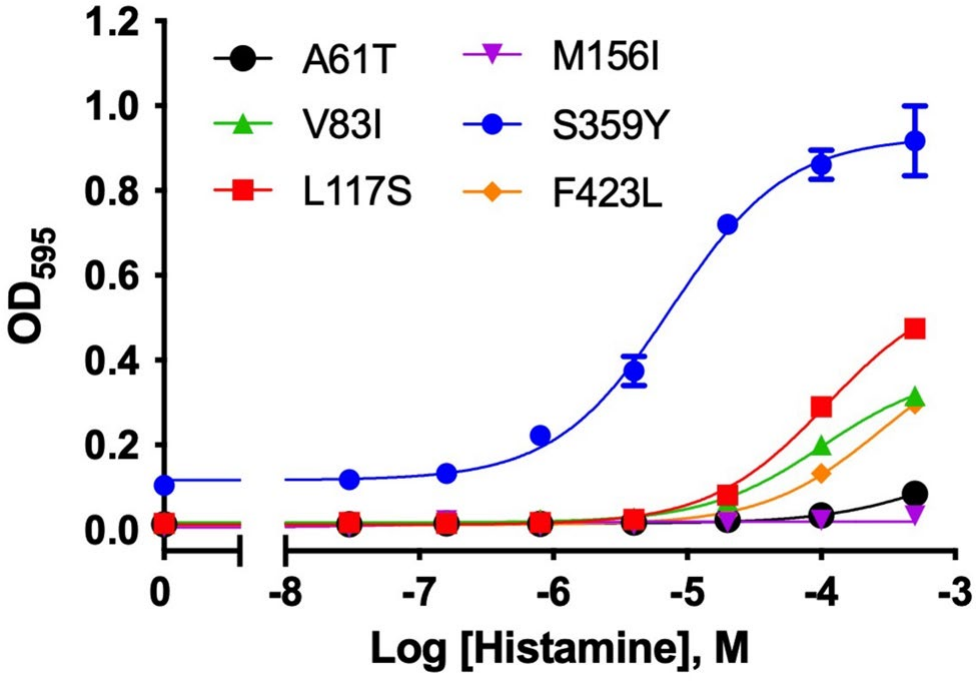
Agonist (histamine) concentration-dependent growth curve of the A61T^1.59^, V83I^2.53^, L117S^3.35^, M156I^4.46^, S359Y^6.36^, and F423L^8.54^ H_3_R mutants. All data shown here are representative of three independent experiments. Each experiment was performed in triplicate. The growth assay data were fitted to a Log (agonist) vs. response model. The average EC_50_ of S359Y was 8.8 ± 2.3 [μM].

Of the clones obtained, r3 (L73M^2.43^), r6 (F193S^ECL2^), and r8 (C415R^7.56^) were single mutants that were important for the recovery of signal transduction activity. Furthermore, C415R^7.56^ was confirmed in two clones, r2 and r8. We opened to focus on clones r1, r2, r5 and r10, in which multiple amino acid substitutions were introduced, and searched for mutations important for activity recovery (Table 2). The location of these mutations was confirmed using an H_3_R homology model registered in the GPCR database (GPCRdb)^32,33^ (Fig. 4a). Ala39^1.37^, Phe102^ECL1^ and Val407^7.48^ pointed to the exterior of the receptor, and the intramolecular interactions seemed to be small. His187^ECL2^, present in the second extracellular loop, located far from a potential ligand-binding site. Thus, mutations at these positions did not seem to significantly alter the receptor function. Therefore, the following six mutations, A61T^1.59^ (clone r5), V83I^2.53^ (r10), L117S^3.35^ (r5), M156I^4.46^ (r10), S359Y^6.36^ (r1), and F423L^8.54^ (r10), were introduced into H3R_i3d alone, and a growth assay was performed. Sufficient growth was only observed with S359Y^6.36^, with low growth activity in the other mutations (Fig. 5). Therefore, S359Y^6.36^ alone could recover signal activity, and a combination of mutations was needed for A61T^1.59^, L117S^3.35^, V83I^2.53^ and F423L^8.54^ to recover the signal activity of clones r5 and r10.

Finally, we identified four mutations, L73M^2.43^, F193S^ECL2^, S359Y^6.36^, and C415R^7.56^ that markedly recovered signal transduction activity. Based on the recently elucidated crystal structure of H_3_R, three mutations, namely L73M^2.43^, S359Y^6.36^, and C415R^7.56^, were present at positions likely to be involved in the movement of TM6 during activation; these mutations were replaced with bulkier (M, Y and R) and/or positively charged (R) side chains (Fig. 4b). F193S^ECL2^ located in the second extracellular loop and faced to a ligand-binding pocket.

### G protein-coupling specificity of mutants with recovered activity

**Figure 6:**
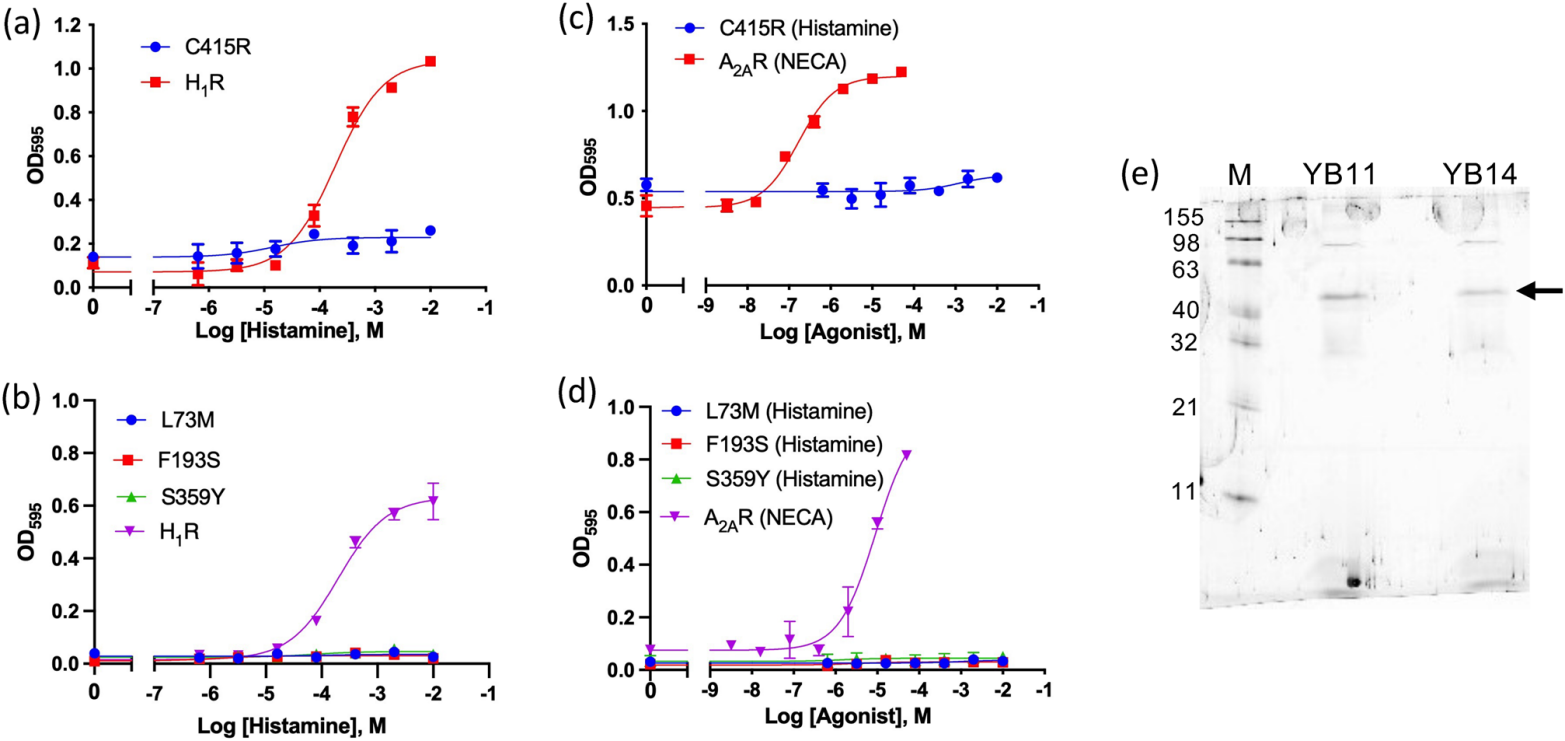
G protein specificity of the H_3_R mutants with recovered activity. (**a**) Growth assay of the C415R mutant in the YB11 strain. (**b**) Growth assay of the L73M, F195S, and S359Y mutants in the YB11 strain. In (**a**) and (**b**), H_1_R is shown as a positive control that couples with G_q_ protein. (**c**) Growth assay of the C415R mutant in the YB14 strain with 20 mM 3-AT. A_2A_R is a positive control. (**d**) Growth assay of the L73M, F195S, and S359Y mutants in YB14 strain with 40 mM 3-AT. In (**c**) and (**d**), A_2A_R is shown as a positive control that couples with G_s_ protein. All data shown are representative of three independent experiments. Each experiment was performed in triplicate. (**e**) In-gel fluorescence analysis after SDS-PAGE of the C415R mutant expressed in YB11 and YB14 cells. Arrow indicates the band derived from H_3_R_i3d-GFP observed between 63 and 40 kDa. *M* molecular size marker.

H_3_R specifically couples to the G_i_ heterotrimeric G protein^12^. To confirm the specificity of the activity-recovered mutants for the G protein, four single mutants (L73M^2.43^, F193S^ECL2^, S359Y^6.36^, and C415R^7.56^) were transformed into the YB11 strain, in which the five C-terminal residues of Gpa1 were replaced with those of human G_q_, and then transformed into the YB14 strain with human G_s_. Thereafter, the growth assay was performed. As positive controls, YB11 transformed with the histamine H_1_ receptor and YB14 transformed with the adenosine A_2A_ receptor grew in an agonist concentration-dependent manner (Fig. 6a–d). YB11 and YB14 transformed with H_3_R mutants did not exhibit histamine-dependent growth (Fig. 6a–d). The expression of H_3_R mutants in YB11 and YB14 was confirmed by in-gel fluorescence after SDS-PAGE (Fig. 6e).

### Evaluation of mutants with recovered activity using agonists and antagonists

**Figure 7:**
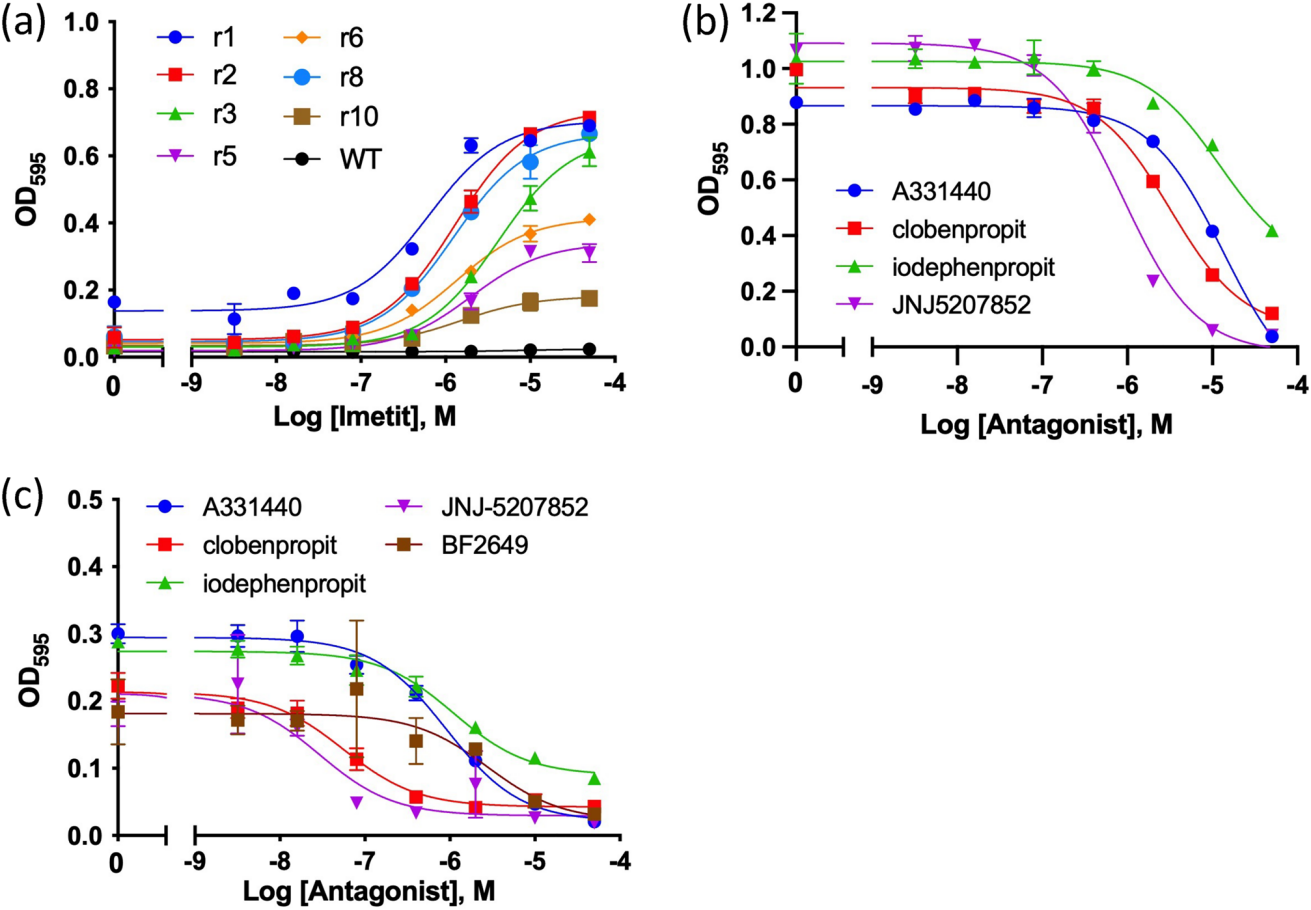
(**a**) Agonist (imetit) concentration-dependent growth assay of wild-type and H_3_R clones with recovered activity. The mean EC_50_ ± SEM is shown in Table 3. (**b**) Antagonist/inverse agonist-concentration dependent inhibition curve of the C415R mutant in the presence of 100 μM histamine. The experiments related to the data in (**a**) and (**b**) were performed in the presence of 20 mM 3-AT. (**c**) Inverse agonist-concentration dependent inhibition curve of the C415R mutant in the absence of histamine. The experiments related to the data in (**c**) were performed in the presence of 10 mM 3-AT. The mean IC_50_ ± SEM values of (**b**) and (**c**) are shown in Table 4. For all experiments, at least three independent experiments were performed, and the representative graph is shown. Each experiment was performed in triplicate. The growth assay data were fitted to a Log (agonist) vs. response model. The inhibition assay data were fitted to a Log (inhibitor) vs. response model.

We evaluated the function of the mutants with recovered activity expressed in YB1 using agonists and antagonists for H_3_R. Synthetic agonist (imetit) and antagonists/inverse agonists (A331440, BF2649, clobenpropit, iodophenpropit, and JNJ-5207852) were used. Imetit is a more potent agonist than histamine^34^. All clones showed agonist concentration-dependent growth of imetit, although wild-type H_3_R_i3d did not show an activity response (Fig. 7a). The maximum growth (OD_595_) was approximately 0.2–0.7, which was generally lower than that of histamine (~ 0.7–1). In particular, the growth of clones r5, r6, and r10 was lower than that of the other clones. This result indicates that these mutations did not completely transmit the structural changes of the receptor to the G protein following imetit binding. The EC_50_ values of the clones with recovered activity were approximately 1/15 those of histamine (Table 3).

**Table 3:** Activation profile of clones with recovered activity by the agonist imetit. EC_50_ values were obtained from the growth assays. The values are expressed as mean ± SD of three independent experiments.

Clone	EC_50_ (μM)
r1	0.92 ± 0.41
r2	0.83 ± 0.33
r3	3.7 ± 0.8
r5	1.7 ± 0.8
r6	1.2 ± 0.2
r8	0.91 ± 0.29
r10	0.89 ± 0.46

Competitive inhibition studies using antagonists A331440, clobenpropit, iodophenpropit, and JNJ-5207852 were performed in the presence of 100 μM histamine (Fig. 7b). Clobenpropit and iodophenpropit are antagonists with imidazole groups, whereas A-331440 and JNJ-5207852 are antagonists without imidazole groups. Each ligand inhibited YB1 cell growth in a concentration-dependent manner by inhibiting histamine binding. The IC_50_ values were markedly higher than the K_i_ values listed in the International Union of Basic and Clinical Pharmacology (IUPHAR) database; however, the magnitude of the IC_50_ was in the order of the K_i_ values (JNJ5207852 < clobenpropit < iodephenpropit, A331440, and BF2649) (Table 4). The inverse agonist activity was examined under conditions of increased constitutive activity by lowering the 3-AT concentration. The inverse agonist (BF2649) and four antagonists (A331440, clobenpropit, iodophenpropit, JNJ-5207852) inhibited cell proliferation in a ligand concentration-dependent manner (Fig. 7c). In the present study, these four antagonists exhibited inverse agonistic activities.

**Table 4:** Inhibition profile of the H_3_R mutant, C415R, with antagonists/inverse agonists in the presence (100 μM) or absence of histamine. IC_50_ values were determined from the inhibition assays shown in Fig. 7. The values are expressed as mean ± SD of three independent experiments. The K_i_ values for human H_3_R were converted from the pK_i_ in the IUPHAR database, (https://www.guidetopharmacology.org).

	K_i_ [nM]	IC_50_ [μM], histamine (+)	IC_50_ [μM], histamine (−)
Clobenpropit	0.40–4.0	7.0 ± 1.5	0.13 ± 0.14
Iodophenpropit	2.0–6.3	24 ± 7	0.64 ± 0.36
A331440	3.2	21 ± 10	2.0 ± 1.6
JNJ-5207852	0.63	1.1 ± 0.5	0.06 ± 0.05
BF2649	2.5–7.9	–	1.6 ± 0.8

## Discussion

*S. cerevisiae* has great potential for the creation of GPCRs with new functions and the elucidation of functional GPCRs. To date, more than 50 GPCRs have been successfully expressed in *S. cerevisiae*; however, many GPCRs remain unsuccessfully expressed^21^. The expression of mammalian GPCRs in yeast appears to have various limitations that must be overcome, such as differences in post-translational processes and membrane lipid composition. Although human H_3_R is expressed in *S. cerevisiae*, no signaling activity was observed in this study. Following random mutagenesis and subsequent in vivo selection with a histamine-containing medium, seven clones with recovered activity that grew in a histamine concentration-dependent manner were selected, and four amino acid mutations (L73M^2.43^, F193S^ECL2^, S359Y^6.36^, and C415R^7.56^) were identified as important for the recovery of activity. Compared with the wild-type, no marked difference in expression was found for the receptor protein in the clones that recovered their activity. A ligand-binding assay using ^3^H-histamine also revealed specific ligand-binding activity in the wild-type. This result suggests that the wild-type receptors were correctly folded and integrated into the cell membrane. Why is the wild-type receptor inactive in *S. cerevisiae*? Although the reason for this inactivity is unknown, we postulate that the wild-type H_3_R is stabilized in an inactive form on the cell membrane of *S. cerevisiae*, resulting in an inactive state.

The L73M^2.43^, S359Y^6.36^, and C415R^7.56^ mutations are located near the DRY (DRF in H_3_R) and NPxxY motifs, which are important for GPCR activation and are commonly found in class A GPCRs. These three mutations are similar as they replace their side chains with bulky chains. L73M^2.43^ is located slightly away from the Gα_i_ binding site. In the crystal structure of the inactive form, Leu73^2.43^ resides in the intracellular region of the second helix and the side chain are 3.8 Å and 3.7 Å away from Tyr412^7.53^ and His416^7.55^ in the NPxxY motif, respectively. Substitution with a larger Met is expected to result in collision with Tyr412^7.53^ and His416^7.55^. This collision may facilitate the transition to an active form. Ser359^6.36^ is present in the intracellular vicinity of TM6, and its side chain forms a hydrogen bond with the main chain of Cys415^7.56^ in the inactive structure. The replacement of Ser359^6.36^ with Tyr is expected to result in collision with Cys415^7.56^. This collision may facilitate the transition to an active state. Cys415^7.56^ is located three residues past the NPxxY motif of TM7 and the side chain points toward TM6. The side chain of Cys415^7.56^ is 4.0 Å away from Val362^6.39^. By replacing Cys415^7.56^ with Arg, collision with Val362^6.39^ is expected, which may facilitate activation. To the best of our knowledge, only few studies have revealed the involvement of the residues at positions 2.43, 6.36, and 7.56 in receptor activity. However, for F193S^ECL2^, it was difficult to speculate the reason for the structure in which the activity was recovered. Detailed mutant analyses and computer simulations are required to understand the roles of these mutations and the mechanisms that facilitate the activation of H_3_R.

In this study, we investigated the reactions of mutants with restored activity when expressed in yeast in the presence of various H_3_R ligands. In a ligand-dependent growth assay, the EC_50_ of the synthetic agonist, imetit, was approximately 11–28 times lower than that of histamine. In addition, the maximal activation of imetit decreased to less than 70% of that of histamine. A calcium mobilization assay using HEK-293 cells also revealed a lower maximal activity of imetit than that of histamine^35^. Maximum imetit activity differed markedly depending on the clone used. Further detailed experiments are required to determine the cause of this phenomenon. According to experiments conducted using the C415R mutant as a representative, all the tested antagonists exhibited inverse agonist activity, as described in a previous review^36^. As described above, the EC_50_ and IC_50_ values were higher than those in animal cells; however, the order of relative H_3_R activity to various ligands was similar to that reported previously. Thus, mutations with recovered activity may retain the characteristics of H_3_R against its ligands.

Receptor stabilization is necessary for the large-scale preparation of proteins for the structural and physicochemical functional analyses of GPCRs. However, stabilizing GPCRs, especially in their active state, remains a challenge. A screening system using *S. cerevisiae* signal transduction would enable efficient production of thermostable mutants while retaining receptor activity. Rag23, a thermally stabilized A_2A_R mutant, retains agonist-induced receptor activity in *S. cerevisiae*, and is thus an example of successful stabilization^7,37^. Recently, we succeeded in stabilizing A_2A_R by combining statistical thermodynamics and molecular evolution^38^. A screening system that uses auxotrophy for selection, as used in this study, is effective for selecting receptor mutants from a vast library of mutants. When examining receptor activity, the time can be shortened by using an assay system with reporter genes, such as GFP or β-galactosidase. In this study, by using yeast, the EC_50_ of histamine binding to the receptor with recovered activity (e.g., for clone r1) was approximately two orders of magnitude higher than that of binding to the wild-type H_3_R analyzed using animal cells^39^. The EC_50_ may be further lowered by increasing sensitivity using tuned yeast strains^26^. Alternatively, sensitivity can be improved by increasing the expression of active receptors by adding a signal sequence or by stabilizing the receptors^40,41^.

In conclusion, random mutagenesis followed by in vivo selection in a histidine-deficient plate medium containing an agonist (histamine) enabled us to obtain mutants of H_3_R with recovered activity, in which the wild-type receptor had no signal transduction activity when expressed in yeast. These mutants retained H_3_R activity in terms of G protein specificity and ligand binding. The strategy for obtaining mutants with recovered activity may enable the recovery of the activity of other GPCRs that do not function in *S. cerevisiae* and may be useful in creating GPCRs mutants stabilized in their active conformations. However, further experiments are needed to elucidate why these mutations recovered the activity.

## Data Availability

The datasets generated in this study are available from the corresponding author upon request.
